# PLK1 inhibition by volasertib suppresses key transcriptional regulators underlying fibroblast activation and pulmonary fibrosis

**DOI:** 10.1152/ajplung.00204.2025

**Published:** 2026-01-02

**Authors:** Priyanka Singh, Pradeep K. Patel, Rajesh K. Kasam, Ryan Lawson, Harshavardhana H. Ediga, Anil G. Jegga, Satish K. Madala

**Affiliations:** 1 Division of Pulmonary, Critical Care and Sleep Medicine, Department of Internal Medicine, University of Cincinnati College of Medicine, Cincinnati, Ohio, United States; 2 Division of Biomedical Informatics, Cincinnati Children’s Hospital Medical Center, Cincinnati, Ohio, United States

**Keywords:** fibroblasts, idiopathic pulmonary fibrosis, MYCN proto-oncogene, Polo-like kinase 1, volasertib

## Abstract

Idiopathic pulmonary fibrosis (IPF) is a progressive and fatal interstitial lung disease marked by aberrant fibroblast activation, resulting in excessive proliferation, survival, and accumulation of extracellular matrix (ECM). A critical barrier to developing effective therapies for IPF is the limited understanding of druggable molecular regulators that control fibroblast activation. In this study, we identify the proto-oncogene MYCN as a key driver upregulated in dysregulated fibroblasts from IPF lungs. Mechanistically, we show that transforming growth factor α (TGFα) induces MYCN expression via the profibrotic transcription factor Wilms’ tumor 1 (WT1). Notably, the knockdown of MYCN significantly attenuated fibroblast proliferation, survival, and ECM production. We further show that MYCN positively regulates the mitotic kinase, Polo-like kinase (PLK1), and that pharmacological inhibition of PLK1 using volasertib reduced expression of MYCN, WT1, and PLK1 and mitigated fibroblast activation. In vivo, volasertib treatment attenuated fibroblast activation and collagen deposition during TGFα-induced pulmonary fibrosis. Together, these findings identify a pathogenic role for the WT1-MYCN-PLK1 axis in fibroblast activation and provide proof-of-concept evidence supporting PLK1 inhibition with volasertib as a potential therapeutic strategy for IPF.

## NEW & NOTEWORTHY

Excessive proliferation, impaired apoptotic clearance, and extracellular matrix (ECM) production are pathological features of fibroblast activation that together result in scar tissue formation in pulmonary fibrosis. This study identifies the previously unrecognized role for the WT1-MYCN-PLK1 axis in promoting fibroblast activation and highlights the therapeutic potential of PLK1 inhibition with volasertib as an antifibrotic strategy.

## INTRODUCTION

Idiopathic pulmonary fibrosis (IPF) is a chronic, progressive interstitial lung disease characterized by excessive proliferation and survival of (myo)fibroblasts, leading to abnormal deposition of extracellular matrix (ECM) proteins in the distal lung and progressive scarring of the lung parenchyma (1, 2). IPF is diagnosed primarily in individuals over the age of 60; it has an estimated global incidence of 1–13 cases per 100,000 persons, with the highest rates reported in South Korea, Canada, and North America (3, 4). Despite the availability of several FDA-approved treatments, the median survival for patients with IPF remains poor, typically ranging from 3 to 5 yr (5–7). Current therapies, nintedanib (Ofev) and pirfenidone (Esbriet), and the recently approved nerandmilast (Jascayd) have been shown to slow the rate of forced vital capacity decline in clinical trials (8–10). Although these drugs differ in their specific molecular targets, all three treatments have been shown to inhibit fibroblast activity and ECM deposition in preclinical models of pulmonary fibrosis (11–13). However, the clinical use of nintedanib and pirfenidone is limited by adverse side effects and patient intolerance, often leading to treatment discontinuation (14–16). Therefore, new therapies are needed that are well-tolerated and can effectively reverse the progression of fibrotic lung disease.

In the pathogenesis of IPF, lung-resident fibroblasts proliferate and differentiate into contractile mesenchymal cells, called myofibroblasts. These cells resist apoptotic clearance and participate in continuous ECM production, secretion, and deposition in distal areas of the lung (17–20). Recent advances in molecular and cellular profiling of the fibrotic lung have identified key signaling pathways and transcriptional regulators that drive this activation. Upon persistent injury and exposure to profibrotic stimuli such as transforming growth factor α (TGFα) and TGFβ, fibroblasts undergo phenotypic changes characterized by increased proliferation, resistance to apoptosis, and excessive ECM production (21, 22). These processes are tightly regulated at the transcriptional level by profibrotic transcription factors, including SMADs, STATs, and zinc finger proteins such as Wilms’ tumor 1 (WT1), which integrate upstream signaling cues to promote fibrogenic gene expression (21–24). Dysregulation of these transcriptional regulators contributes to sustained fibroblast activation. Studies from our laboratory and others have shown an upregulation of WT1 in both mesenchymal and mesothelial cells that accumulate in the distal regions of fibrosing lungs in IPF and mouse models of pulmonary fibrosis. Our gain-of-function and loss-of-function studies suggest that WT1 functions as a positive regulator of proliferation, fibroblast-to-myofibroblast transformation, survival, and ECM production (17, 19, 23, 25, 26). Transcriptomic profiling of WT1-deficient fibroblasts identified multiple downstream effectors, including MYCN, that further amplify pathogenic gene networks involved in cell cycle progression, survival, and ECM production (17, 22, 25). Targeting transcriptional regulators that drive these core profibrotic programs represents a promising therapeutic strategy to halt or reverse the fibrotic response in IPF and related interstitial lung diseases.

MYCN is a member of the MYC family of transcription factors and plays an important role in signaling pathways involved in cell growth. Although its role in cancer is well-documented, its function in pathological fibroblast activation and pulmonary fibrosis remains poorly understood. Importantly, MYCN regulates Polo-like kinase (PLK1), a key cell cycle regulator (27–29). Recent studies suggest that PLK1 functions as a positive regulator of MYCN. Stabilized MYCN, in turn, activates PLK1 transcription, establishing a positive feedback loop that amplifies MYCN-driven transcriptional programs involved in cell growth (27, 28). However, the role of the MYCN-PLK1 axis in the context of WT1-driven fibroblast activation remains largely unknown.

The current study focuses on the complex interactions between WT1, MYCN, and PLK1. Our results showed, for the first time, that MYCN is upregulated in the lungs and fibroblasts of patients with IPF and a mouse model of TGFα-induced pulmonary fibrosis. We demonstrated that WT1 acts as a key transcription factor for TGFα-induced MYCN expression in fibroblasts. Notably, knockdown of MYCN attenuated PLK1 expression and fibroblast activation, including proliferation, survival, and ECM production. Furthermore, a known PLK1 inhibitor, volasertib, attenuated the expression of PLK1, MYCN, and WT1, effectively mitigating the profibrotic activity of IPF fibroblasts. Finally, in vivo studies demonstrate that volasertib reduces fibroblast activation and ECM deposition, providing important proof-of-concept data for the use of volasertib in treating pulmonary fibrosis.

## MATERIALS AND METHODS

### Human Samples

IPF and healthy donor lung samples were obtained with assistance from the Translational Pulmonary Science Center at the University of Cincinnati Medical Center. This center maintains a repository of tissue and primary cells from patients with chronic lung diseases. All human IPF (*n* = 6 male) and normal control lung explants (*n* = 5 male, *n* = 1 female) used in this study were predominantly from male donors aged 50–75 yr. Lung explants used as controls were obtained from donor lungs that exhibited no evidence of lung disease and were considered unsuitable for transplantation, including size mismatch, logistical constraints, or accidental death. IPF lung explants were collected from individuals undergoing therapeutic lung transplantation. Procedures for collecting and maintaining samples are approved by the University of Cincinnati Institutional Review Board (IRB; Protocol No. 2013-8157), ensuring the protection of human subjects’ privacy and confidentiality in compliance with HIPAA regulations.

### Mouse Model of TGFα-Induced Pulmonary Fibrosis

We used a transgenic mouse model of pulmonary fibrosis that overexpresses TGFα on the FVB/NJ inbred strain background, as previously described (30). Homozygous CCSP^rtTA^ mice were crossed with heterozygous TetO-cmv-TGFα mice to generate bitransgenic TGFα^OE^ (CCSP/TGFα) mice and littermate controls (CCSP^/−^). Both male and female mice aged 12–16 wk were used for the experiments. TGFα expression was induced by administering doxycycline (DOX; 62.5 mg/kg) through food and water, resulting in severe fibrotic lung disease after 4 wk of treatment (31). All mice were housed under specific pathogen-free conditions at the University of Cincinnati. For in vivo experiments, volasertib (Selleckchem, Cat. No. S2235) was freshly formulated in 0.5% carboxy methyl cellulose (Sigma, Cat. No. C5678) in PBS. Mice were anesthetized with isoflurane before drug administration. For oral administration, 10 mg/kg dosage of volasertib was used for each mouse on every other day for 4 wk.

### Histology and Immunohistochemistry

Histological procedures were performed, as mentioned previously (31). In brief, paraffin-embedded tissues were cut into 5-μm sections and deparaffinized for Masson’s trichrome staining. For immunohistochemistry, lung sections were deparaffinized, followed by citric acid antigen retrieval (pH 6.0) and methanol treatment, blocked in 5% donkey serum, incubated with the primary antibody overnight, incubated with species-specific secondary peroxidase antibodies, and then stained with diaminobenzidine. Isotype control antibodies matching the host species and the IgG subclass of the primary antibodies were used to confirm the specificity of immunohistochemical staining. Antibody details are provided in Table 3. Bright-field images were captured using a Keyence BZ series microscope and analyzed using BZ-X Analyzer software (Keyence Corporation, Osaka, Japan). Immunohistochemistry was performed simultaneously using slides from the control and treatment groups. All images were acquired using identical imaging settings to ensure consistency across samples. Image acquisition was performed randomly across comparable anatomical regions, and subsequent analyses were conducted in a blinded manner to minimize experimental bias.

### Hydroxyproline Content Estimation

Total lung hydroxyproline content was measured using a colorimetric assay (Abcam, Cat. No. ab222941), following the manufacturer’s protocol and as previously described (32). In brief, lung tissue was hydrolyzed in 10 N sodium hydroxide (NaOH), followed by neutralization with 10 N hydrochloric acid (HCl). The released hydroxyproline was then oxidized to form a pyrrole intermediate, which subsequently reacted with Ehrlich’s reagent in perchloric acid to produce a chromogenic complex. Absorbance was measured at 560 nm, and hydroxyproline concentrations were determined based on a standard curve.

### RNA Preparation and Real-Time PCR

RNA was isolated from lung tissue homogenates using an RNAeasy mini kit (Qiagen, Cat. No. 74106) following tissue homogenization with beads and a high-speed homogenizer (Qiagen, TissueLyzer II), as previously described (33). cDNA synthesis was performed using Superscript III (Invitrogen, Cat. No. 56575), and real-time PCR was conducted using SYBR Green Select Master Mix (Applied Biosystems, Cat. No. 4309155) in CFX Opus 384 Touch Real-Time PCR System (Bio-Rad Laboratories, Hercules, CA). Target gene expression was normalized to β-actin for human samples and hypoxanthine-guanine phosphoribosyltransferase (HPRT) for mouse samples (Tables 1 and 2). For each gene and experimental run, a single control replicate was designated as the calibrator (assigned a value of 1); consequently, the remaining control replicates were not constrained to 1, allowing for the assessment of biological variance within the control group.

### Primary Fibroblast Cultures and Treatments

Primary fibroblasts were isolated from the distal subpleural fibrotic parenchyma of the right lower lobe from both patients with IPF and control donor lungs. Tissue was processed via collagenase digestion, followed by the depletion of hematopoietic cells through negative selection for CD45 using MACS (human CD45 microbeads, Cat. No. 130-045-801, Miltenyi Biotec), as previously described (31). Similarly, mouse fibroblasts were negatively selected for CD45 using MACS (mouse CD45 microbeads, Cat. No. 130-052-301, Miltenyi Biotec). Fibroblasts were cultured in DMEM supplemented with 10% FBS and antibiotics for human or IMDM supplemented with 5% FBS for mouse. The human fibroblasts of passages 1 to 4 were used for all in vitro experiments. Cells were treated with TGFα (100 ng/mL) (PeproTech, Cat. No. 100-16A) overnight at low serum conditions. Fibroblasts were treated with vehicle (DMSO) or volasertib for 48 h. For siRNA-mediated knockdown studies, IPF fibroblasts were transfected with control or MYCN-specific siRNA from Invitrogen for 72 h using a Lipofectamine 3000 transfection kit (Life Technologies, Cat. No. L3000015), as described previously (34). Each sample (*n*) represents a biological replicate, defined as an independently derived fibroblast culture from a unique donor lung.

### Western Blot

Western blotting was performed, as previously described (35). The membranes were blocked with 5% BSA in 0.1% Tween phosphate-buffered saline and incubated overnight with primary antibodies at 4°C. Protein bands were visualized using ECL Prime Western Blotting Detection Reagent (Thermo Fisher, Cat. No. RPN2232) in ChemiDoc MP Imaging System (Bio-Rad Laboratories, Hercules, CA) according to the manufacturer’s instructions. All immunoblot images were processed and analyzed using Image Lab software (version 6.1, Bio-Rad). Protein expression levels were quantified and normalized to GAPDH, which served as a loading control. Normalized values were expressed as the ratio of the target protein to GAPDH. Details of all antibodies used in this study, including target, source, and dilution, are provided in Table 3. Full blot images are shown in Supplemental Figs. S2–S5.

### Quantification of Apoptosis

Quantification of apoptotic cell death was measured either by using the Caspase 3/7 Green apoptosis assay or the Tunnel assay. Apoptotic cell death measurement using Caspase 3/7 activity was described previously using the IncuCyte ZOOM real-time imaging system (31). In brief, primary cells were incubated in Caspase 3/7 Green apoptosis assay reagent (Caspase 3/7 substrate conjugated to green fluorophore; Essen BioScience). Time-lapse fluorescence imaging was performed using the IncuCyte ZOOM system (Essen BioScience, Ann Arbor, MI); nine images per well were collected at ×20 magnification every 2 h for 14 h. The average number of green objects produced by the apoptotic cells was measured using IncuCyte ZOOM software 2015 A (Essen BioScience, Ann Arbor, MI). For the tunnel assay, human IPF fibroblasts were seeded at 3 × 10^4^ cells/well in an eight-well chambered slide and incubated overnight. Cells were then treated with either vehicle or volasertib for 24 h. Human-specific anti-Fas antibody (250 ng/mL, Millipore Sigma) was added along with vehicle or volasertib and further incubated for 24 h. Nuclei were stained using DAPI, and staining of apoptotic cells was performed using the terminal deoxynucleotidyl TUNEL method using an In Situ Cell Death Detection Kit, TMR Red (Roche Diagnostics, Cat. No. 12156792910). All images were captured at ×20 magnification using a Keyence BZ series microscope (Keyence Corporation, Osaka, Japan) and quantified using a BZ-X Analyzer software (Keyence). Image acquisition and analysis were performed randomly and in a blinded manner to minimize bias.

### BrdU Incorporation Assay

Cell proliferation assay was performed using a BrdU incorporation assay kit (Cat. No. 6813S, Cell Signaling Technology, Denver, CO), following the manufacturer’s protocol, and as previously described (19). Lung-resident fibroblasts were isolated and cultured for 48 h under low serum conditions (0.5% FBS) in 96-well plates. After the initial 24 h, cells were pulsed with a BrdU labeling solution and incubated for an additional 24 h. Following incubation, the cells were fixed, and the DNA was denatured to expose incorporated BrdU. Proliferation was quantified by incubating with a mouse anti-BrdU monoclonal antibody, followed by a horseradish peroxidase-conjugated secondary antibody and TMB substrate development. The absorbance at 450 nm was measured, which is directly proportional to the magnitude of BrdU incorporation and DNA synthesis.

### Statistics

All data were analyzed using GraphPad Prism (version 10.5; GraphPad Software, San Diego, CA). Statistical tests were selected based on the distribution characteristics of the data and the experimental design. Depending on the comparison, data were analyzed using the Student’s two-tailed *t* test, one-way ANOVA or two-way ANOVA. The specific statistical test used is indicated in each figure legend. Exact *P* values are reported to denote statistical significance. All data are presented as means ± standard deviation (SD). All measurements were performed in a blinded manner to minimize experimental bias.

### Ethics Statement

Human lung tissue samples were collected and used in accordance with the University of Cincinnati Institutional Review Board (IRB) guidelines (Protocol No. 2013-8157), ensuring participant protection and confidentiality. All animal experiments were performed in an AAALAC-accredited, specific pathogen-free facility at the University of Cincinnati, under protocols approved by the Institutional Animal Care and Use Committee (IACUC).

## RESULTS

### MYCN Is Upregulated in Fibroblasts during Pulmonary Fibrosis

**Figure 1.**
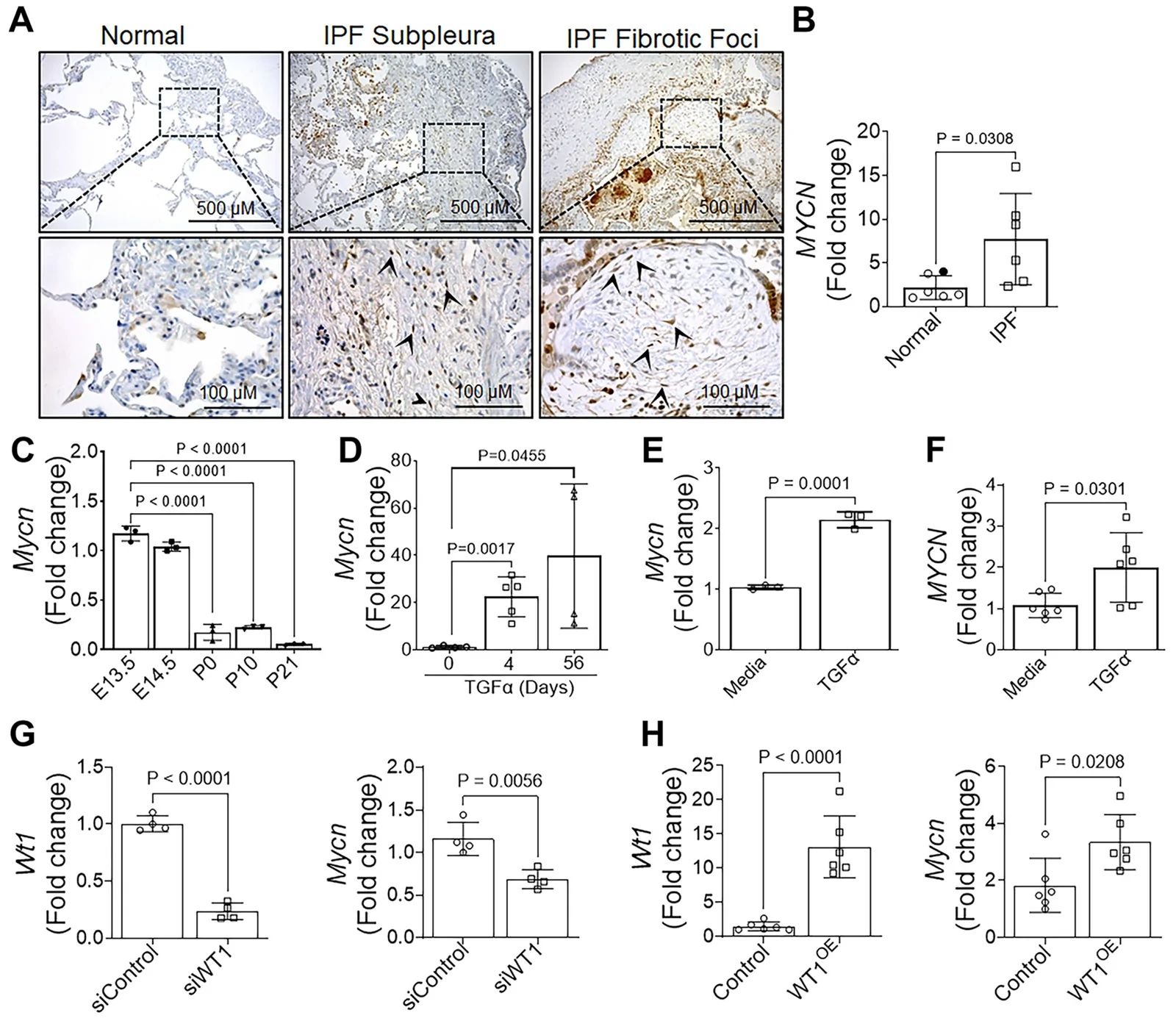
MYCN upregulation in fibroblasts during pulmonary fibrosis. *A*: representative images of distal lung sections from normal and IPF lungs immunostained with anti-MYCN. Upper panels represent the low magnification images (×10), whereas the lower panel represents high magnification images (×40). Arrowheads highlight MYCN nuclear staining. *B*: quantification of *MYCN* gene transcripts by RT-PCR in fibroblasts isolated from either normal (*n* = 5 male, *n* = 1 female) or IPF (*n* = 6 male) donor lung tissue (Student’s 2-tailed *t* test). *C*: quantification of Mycn gene transcripts at different stages of mouse lung development by RT-PCR (*n* = 3–7 mice lungs/group; 1-way ANOVA followed by Tukey’s post hoc test). *D*: quantification of *Mycn* gene transcripts using RT-PCR in total lung RNA isolated from TGFα overexpression mice on doxycycline food for 4 and 8 wk (*n* = 4 or 5 mice lungs/group; Student’s 2-tailed *t* test). *E*: lung-resident fibroblasts isolated from normal murine lungs were treated with media or TGFα for 24 h, and *Mycn* transcripts were quantified by RT-PCR (*n* = 3 mice lungs/group; Student’s 2-tailed *t* test). *F*: quantification of *MYCN* gene transcripts by RT-PCR in human fibroblasts from healthy donor lung tissue treated with either media or TGFα for 24 h (*n* = 6 donor samples/group; Student’s 2-tailed *t* test). *G*: lung-resident fibroblasts from TGFα mice on DOX for 4 wk were transiently transfected with control or WT1 siRNA for 72 h, and transcripts of *Mycn* and *Wt1* were quantified using RT-PCR (*n* = 4 mice/group; Student’s 2-tailed *t* test). *H*: lung-resident fibroblasts from normal control mice were transduced with control or WT1 overexpressing lentivirus for 72 h, and transcripts of *Mycn* and *Wt1* were quantified using RT-PCR (*n* = 6 mice/group; Student’s 2-tailed *t* test). IPF, idiopathic pulmonary fibrosis; TGFα, transforming growth factor α; WT1, Wilms’ tumor 1.

To determine whether MYCN is upregulated in the lungs of IPF, we immunostained lung sections with an antibody against MYCN and observed a marked increase in MYCN staining in spindle-shaped fibroblasts located in fibroblastic foci and subpleural regions of IPF lung tissue compared with normal donor lungs (Fig. 1A and Supplemental Fig. S1). Also, we quantified MYCN transcript levels in lung-resident fibroblasts isolated from IPF and control donor lungs and observed a significant increase in MYCN transcripts in IPF fibroblasts compared with fibroblasts from normal lungs (Fig. 1B).

MYCN is highly expressed in lung development and plays a critical role during embryonic lung development (6, 7). We assessed the kinetics of MYCN expression during lung development and postnatal stages of lung development in mice. As expected, we observed high levels of *Mycn* transcripts in the early stages of lung development, and subsequently declined, with the lowest expression observed in the adult lungs (Fig. 1C). To determine whether MYCN is upregulated during pulmonary fibrosis in adult lungs, we quantified *Mycn* transcripts in total lung RNA of mice during TGFα-induced pulmonary fibrosis. We observed a significant increase in *Mycn* transcript levels in the lungs during TGFα-induced pulmonary fibrosis (Fig. 1D). To determine the direct effects of TGFα on MYCN expression, we have quantified *Mycn* transcripts in lung-resident fibroblasts treated with media or TGFα for 16 h and observed a significant increase in *Mycn* transcripts with TGFα treatment compared with media in fibroblasts from mouse lungs (Fig. 1E). Consistently, we observed a significant increase in *MYCN* transcript levels with TGFα treatment in fibroblasts isolated from normal human donor lungs (Fig. 1F).

TGFα promotes pulmonary fibrosis by activating epidermal growth factor receptor signaling, which induces the profibrotic transcription factor WT1, leading to sustained fibroblast activation and ECM deposition (21, 30). To assess whether WT1 promotes MYCN expression, fibroblasts isolated from fibrotic lung lesions of TGFα overexpressing mice were treated with control or WT1-specific siRNA for 72 h, and the knockdown of WT1 was sufficient to reduce *Mycn* transcripts level (Fig. 1G). Conversely, overexpression of WT1 using adenovirus resulted in a significant increase in *Mycn* transcripts in lung-resident fibroblasts isolated from normal mice (Fig. 1H). Together, these new findings suggest that MYCN is upregulated in fibroblasts via the TGFα-WT1 axis in the pathogenesis of pulmonary fibrosis.

### MYCN Functions as a Positive Regulator of ECM Gene Expression in IPF Fibroblasts

**Figure 2.**
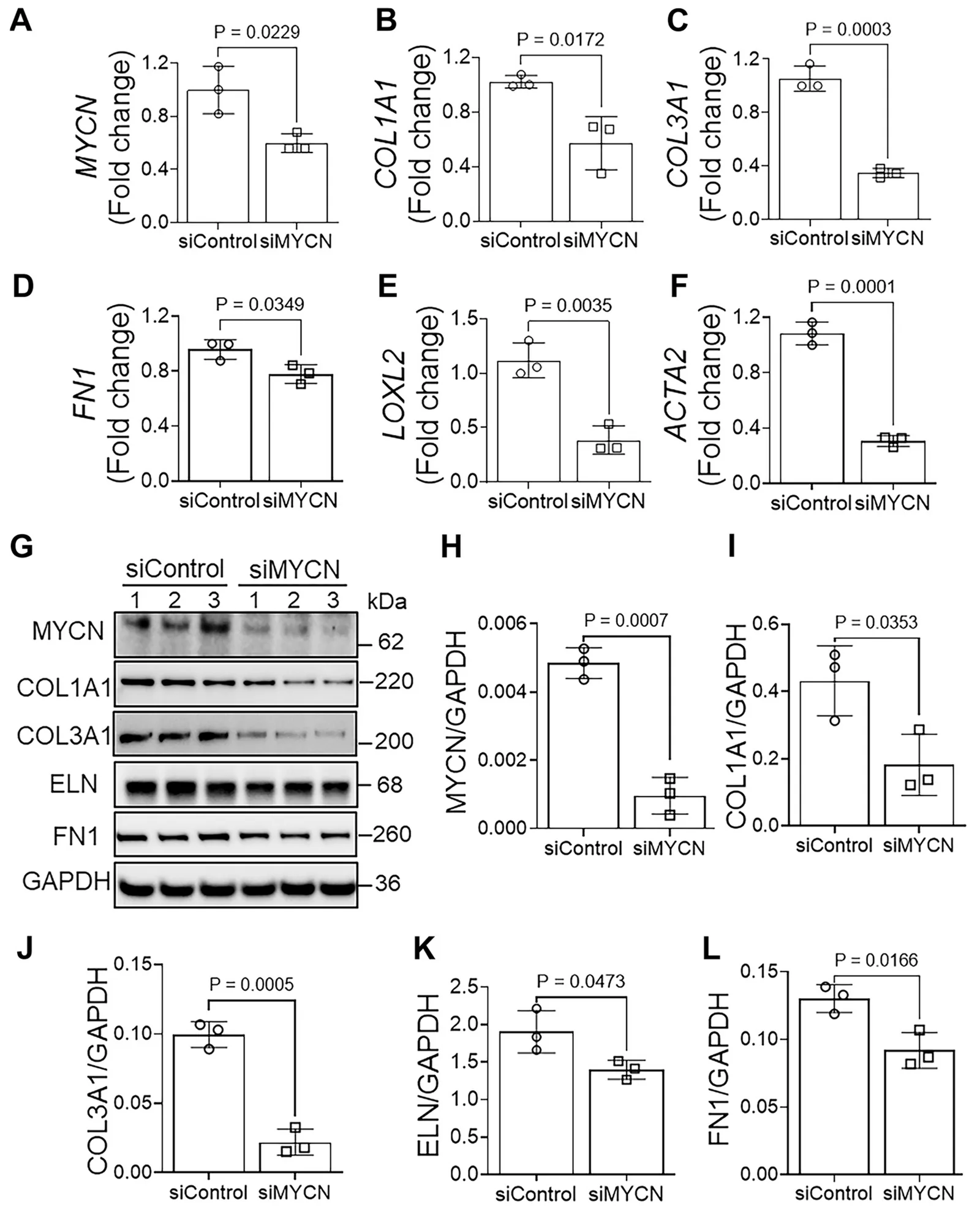
MYCN is a positive regulator of ECM gene expression in IPF fibroblasts. *A–F*: quantification of *MYCN, COL1A1, COL3A1, FN1, LOXL2*, and *ACTA2* gene transcripts by RT-PCR in IPF fibroblasts treated with either control or MYCN-specific siRNA for 72 h (*n* = 3 donor samples/group; Student’s 2-tailed *t* test). *G*: IPF fibroblasts were treated with either control or MYCN-specific siRNA for 72 h, and cell lysates were immunoblotted with antibodies against MYCN, COL1A1, COL3A1, ELN, FN1, and GAPDH. *H–L*: MYCN, COL1A1, COL3A1, ELN, and FN1 protein levels were normalized to GAPDH (*n* = 3 donor samples/group; Student’s 2-tailed *t* test). ECM, extracellular matrix; IPF, idiopathic pulmonary fibrosis.

The activated fibroblast, particularly the myofibroblast, is the major cell type responsible for collagen deposition, including collagen types I and III, contributing to the scarring and stiffening of lung tissue in severe fibrotic lung disease (8, 9). Given that WT1 regulates the expression of both MYCN and ECM genes, we assessed whether MYCN alters ECM gene expression in IPF fibroblasts (2). Notably, the knockdown of MYCN in fibroblasts isolated from the lungs of patients with IPF was sufficient to attenuate expression of ECM genes, including *COL1A1, COL3A1, FN1, LOXL2*, and *ACTA2* (Fig. 2, A–F). We next confirmed the knockdown effects of MYCN on ECM protein levels through Western blot analysis of IPF fibroblasts treated with MYCN-specific siRNA compared with those treated with control siRNA for 72 h. Consistent with changes in gene transcripts, we observed a significant decrease in ECM-associated protein levels (COL1A1, COL3A1, FN1, and ELN) with the loss of MYCN in IPF fibroblasts (Fig. 2, G–L). In summary, these results indicate positive regulation of ECM production and remodeling by MYCN in IPF fibroblasts.

### MYCN Promotes the Proliferation and Survival of IPF Fibroblasts

**Figure 3.**
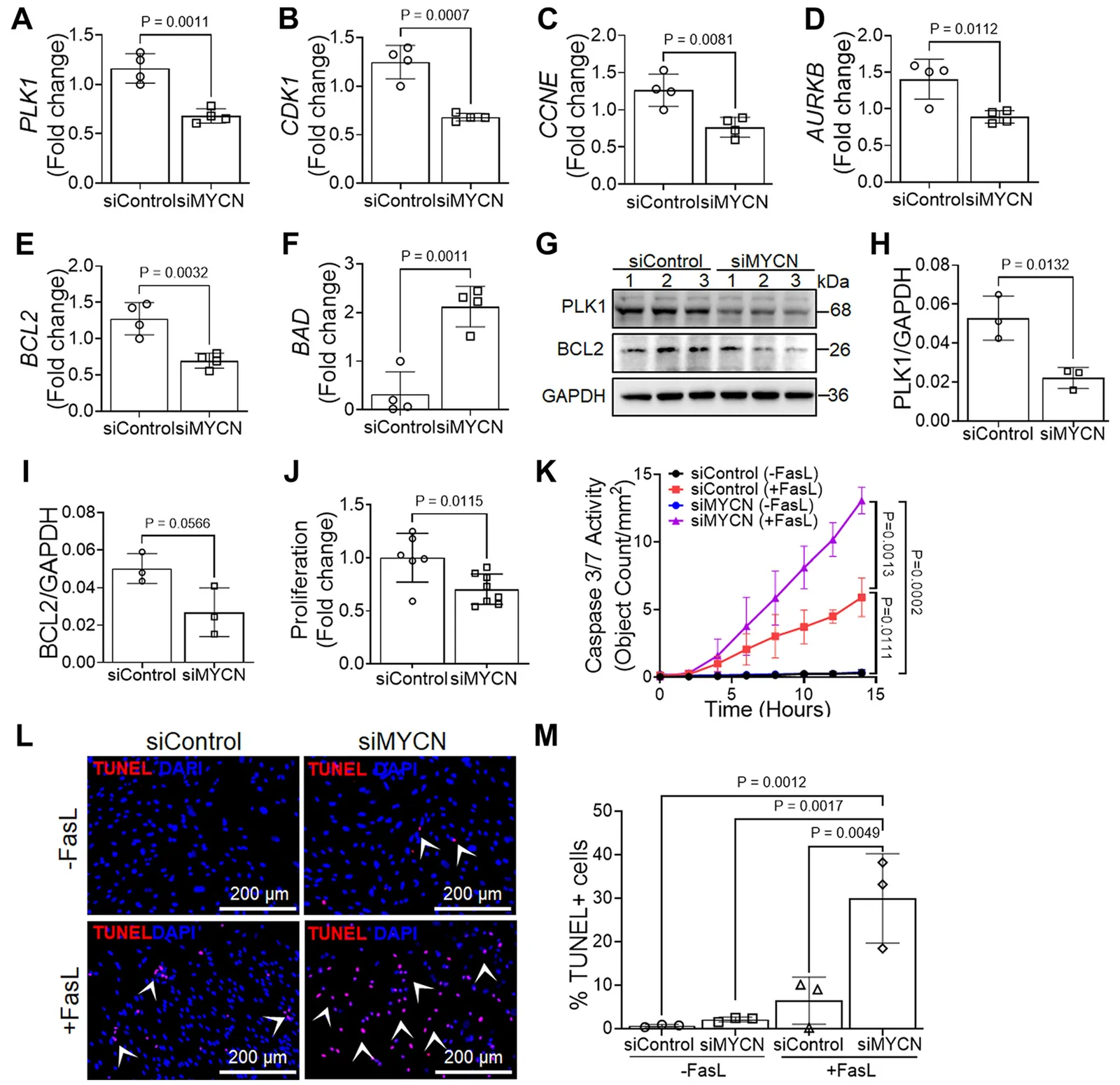
The knockdown of MYCN attenuates proliferation and survival in IPF fibroblasts. *A–F*: quantification of proliferation (*PLK1, CDK1, CCNE1*, and *AURKB*) and survival (*BCL2* and *BAD*) gene transcripts by RT-PCR in IPF fibroblasts treated with either control or MYCN-specific siRNA for 72 h (*n* = 4 donor samples/group; Student’s 2-tailed *t* test). *G*: IPF fibroblasts were treated with either control or MYCN-specific siRNA for 72 h, and cell lysates were immunoblotted with antibodies against PLK1, BCL2, and GAPDH. *H* and *I*: PLK1 and BCL2 protein levels were normalized to GAPDH (*n* = 3 donor samples/group; Student’s 2-tailed *t* test). *J*: proliferation was assessed using BrdU incorporation assay in IPF fibroblasts treated with either control or MYCN-specific siRNA (*n* = 6–8/group; Student’s 2-tailed *t* test). *K*: quantification of apoptotic cells (Caspase 3/7-positive cells) using IncuCyte ZOOM in IPF lung fibroblasts treated with control or MYCN-specific siRNA for 72 h (*n* = 4 donor samples/group; 2-way ANOVA followed by Tukey’s post hoc test). *L*: IPF fibroblasts were treated with either control siRNA or MYCN-specific siRNA for 72 h in the presence and absence of anti-Fas for 24 h. Cells were stained to quantify TUNEL-positive cells using fluorescence microscopy. Representative images were collected at ×20 magnification. Scale bar, 200 μm. Arrowheads highlight TUNEL-positive cells. *M*: quantification of TUNEL-positive cells in IPF fibroblasts treated with either siControl or siMYCN (*n* = 3 donor samples/group; 1-way ANOVA followed by Tukey’s post hoc test). FasL, Fas ligand; IPF, idiopathic pulmonary fibrosis.

To determine whether MYCN contributes to fibroblast activation, we treated IPF fibroblasts with control or MYCN-specific siRNA for 72 h and quantified transcripts using real-time PCR for proliferation genes (*PLK1, CDK1, CCNE1*, and *AURKB*) and survival genes (*BCL2* and *BAD*) (Fig. 3, A–F). The loss of MYCN resulted in a significant decrease in gene transcripts associated with fibroblast proliferation and survival (*PLK1, CDK1, CCNE1, AURKB*, and *BCL2*), as well as an increase in proapoptotic gene *BAD* transcripts (Fig. 3, A–F). We next confirmed the knockdown effects of MYCN on PLK1 and BCL2 protein levels through Western blot analysis of IPF fibroblasts treated with MYCN-specific siRNA compared with those treated with control siRNA for 72 h. Consistent with changes in gene transcripts, we observed a decrease in PLK1 and BCL2 protein levels with the loss of MYCN in IPF fibroblasts (Fig. 3, G–I).

To test whether MYCN induces fibroblast proliferation, we assessed the impact of MYCN knockdown on BrdU incorporation in fibroblasts isolated from the lungs of IPF. We observed a significant decrease in BrdU incorporation in fibroblasts treated with MYCN-specific siRNA compared with control siRNA (Fig. 3J). These findings establish that MYCN functions as a positive regulator of fibroblast proliferation in IPF.

In progressive fibrosis, fibroblasts and myofibroblasts develop resistance to apoptosis, thereby promoting excessive ECM deposition and the expansion of fibrotic lung lesions (11, 41). To test whether MYCN induces fibroblast survival, the mature fibrotic lesions of IPF were cultured to isolate fibroblasts, and apoptosis was quantified in fibroblasts treated with either control or MYCN-specific siRNA. Treatment of Fas ligand (FasL) agonist significantly induced apoptosis in IPF fibroblasts. Notably, the loss of MYCN transcripts in the presence of FasL agonist further enhanced the apoptosis induction in fibroblasts from IPF (Fig. 3, K–M). Together, the above findings establish that increased MYCN expression contributes to proliferation and apoptosis resistance in IPF fibroblasts.

### Volasertib Inhibits the Proliferation and Survival of IPF Fibroblasts

**Figure 4.**
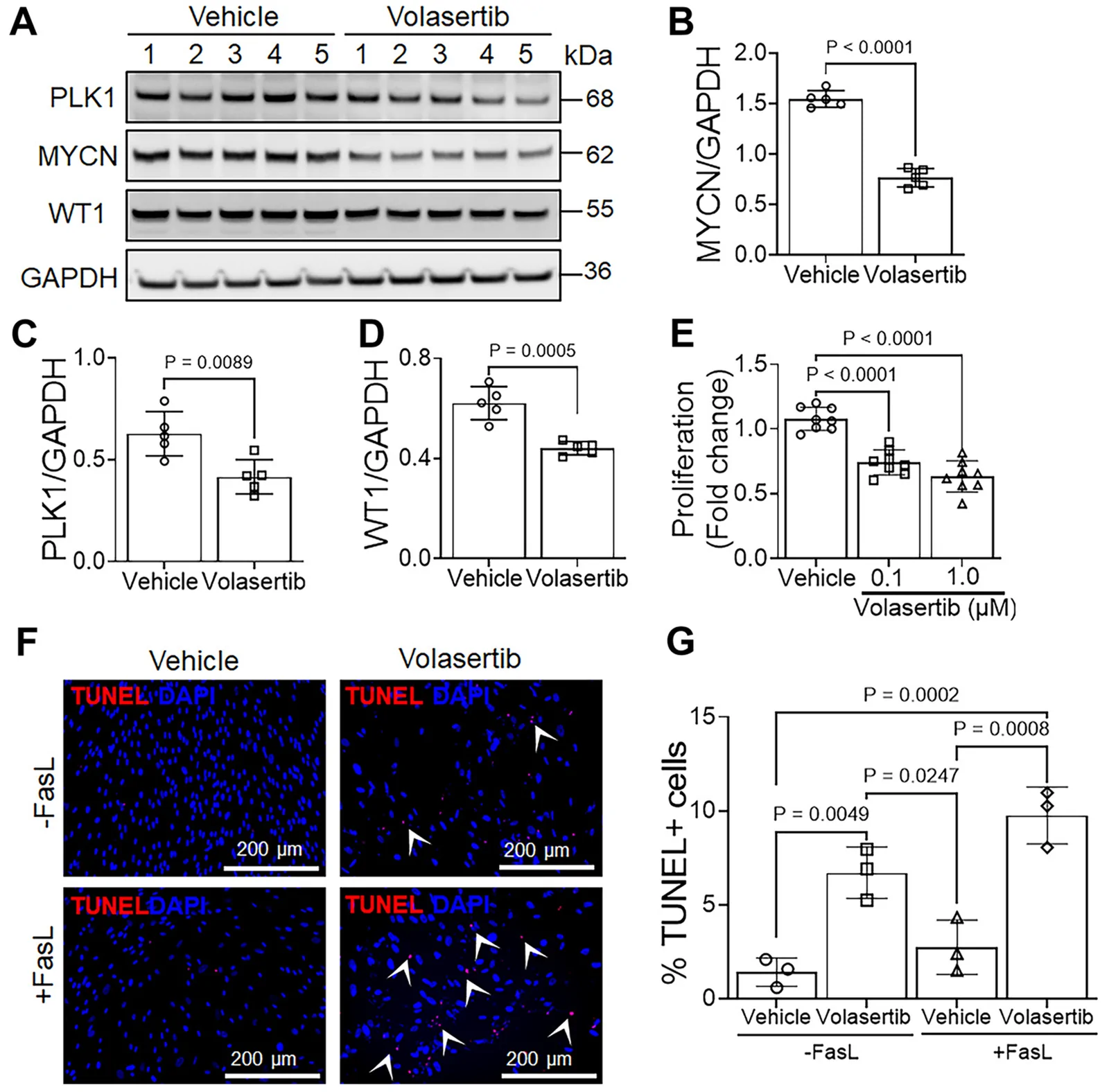
Volasertib inhibits proliferation and survival of IPF fibroblasts. *A*: IPF lung fibroblasts were treated with either vehicle or volasertib (1 μM) for 48 h, and cell lysates were immunoblotted with antibodies against PLK1, MYCN, WT1, and GAPDH. *B–D*: MYCN, PLK1, and WT1 protein levels were normalized to GAPDH (*n* = 5 donor samples/group; Student’s 2-tailed *t* test). *E*: IPF fibroblasts were treated with either vehicle or volasertib (0.1 and 1 μM) for 24 h, followed by BrdU treatment for 12 h (*n* = 8/group; 1-way ANOVA followed by Tukey’s post hoc test). *F*: IPF fibroblasts were treated with either vehicle or volasertib (1 μM) specific for 48 h, in the presence and absence of anti-Fas treatment for 24 h, and cells were stained to quantify total TUNEL-positive cells using fluorescence microscopy. Representative images were collected at ×20 magnification. Scale bar, 200 μm. Arrowheads highlight TUNEL-positive cells. *G*: quantification of TUNEL-positive cells in IPF fibroblasts treated with either vehicle or volasertib (*n* = 3 donor samples/group; 1-way ANOVA followed by Tukey’s post hoc test). FasL, Fas ligand; IPF, idiopathic pulmonary fibrosis; WT1, Wilms’ tumor 1.

To test whether the inhibition of PLK1 activity alters MYCN expression, we assessed the effects of volasertib treatment on the expression of PLK1 and MYCN in fibroblasts isolated from the lungs of IPF. Upon treatment with volasertib, a known PLK1 inhibitor, we observed a significant reduction in the expression of PLK1 and MYCN (Fig. 4, A–C). Volasertib treatment also reduced WT1 protein levels in IPF fibroblasts (Fig. 4, A and D). Similar to the loss of MYCN expression, inhibition with volasertib reduced the proliferation of IPF fibroblasts (Fig. 4E). To assess the effect of volasertib on fibroblast survival, we treated fibroblasts isolated from IPF lungs with volasertib in the presence and absence of FAS ligand and evaluated the percentage of TUNEL-positive apoptotic cells. Treatment with volasertib resulted in a significant increase in TUNEL-positive cells compared with vehicle control (Fig. 4, F and G). Thus, either the loss of MYCN expression or inhibition of PLK1 with volasertib is sufficient to mitigate fibroblast activation, including proliferation and survival in IPF fibroblasts.

### Volasertib Inhibits Fibroblast Activation and Pulmonary Fibrosis

**Figure 5.**
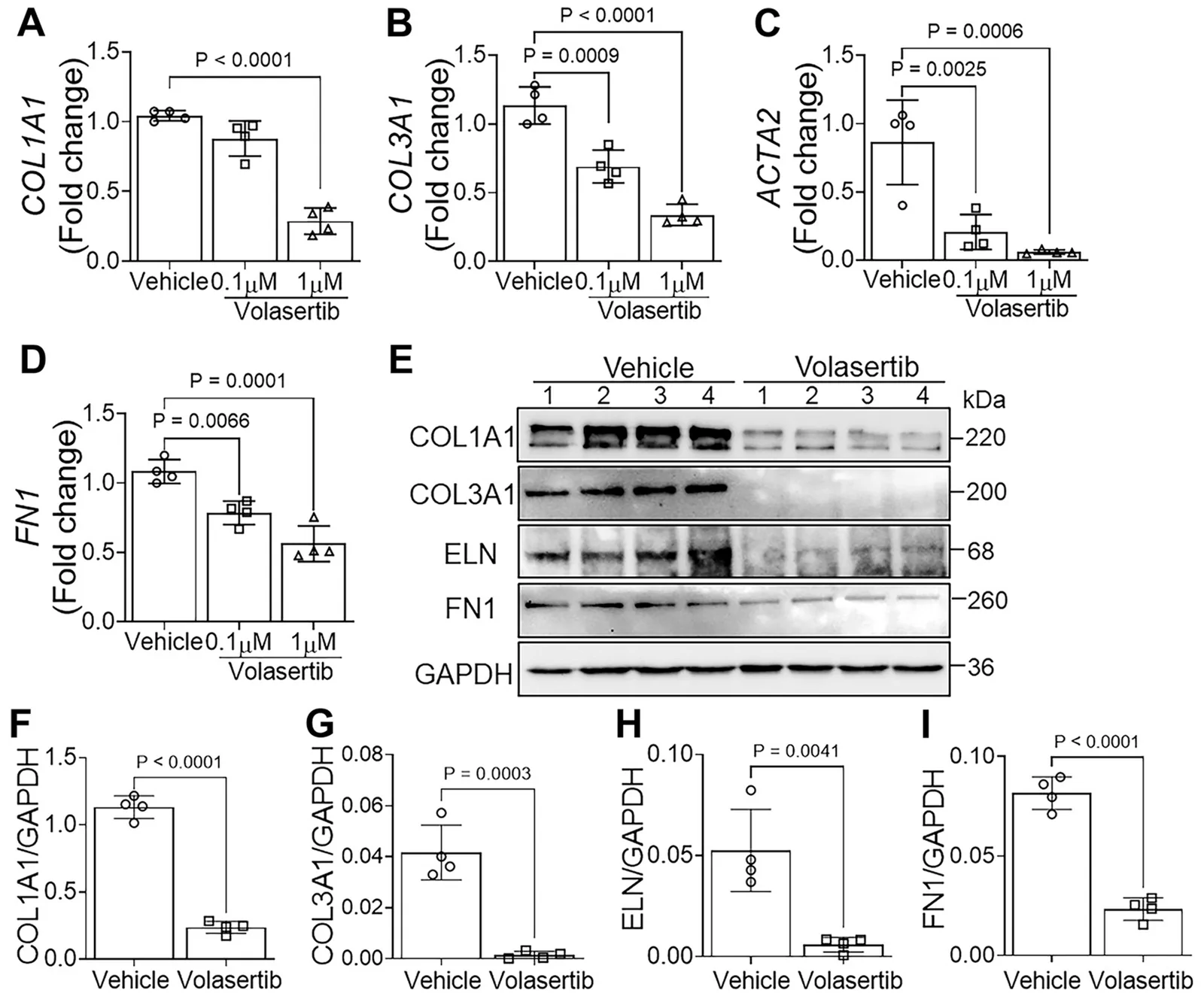
Volasertib inhibits ECM production in IPF fibroblasts. *A–D*: quantification of *COL1A1, COL3A1, ACTA2,* and *FN1* gene transcripts in IPF fibroblasts upon treatment with either vehicle or volasertib (0.1 and 1 μM) for 24 h (*n* = 4 donor samples/group; 1-way ANOVA followed by Tukey’s post hoc test). *E*: IPF lung fibroblasts were treated with either vehicle or volasertib (1 μM) for 48 h, and cell lysates were immunoblotted with antibodies against COL1A1, COL3A1, ELN, FN1, and GAPDH. *F–I*: densitometric quantification for COL1A1, COL3A1, ELN, and FN1 protein levels normalized with GAPDH (*n* = 5 donor samples/group; Student’s 2-tailed *t* test). ECM, extracellular matrix; IPF, idiopathic pulmonary fibrosis.

To assess the effect of volasertib on ECM gene expression, IPF fibroblasts were treated with vehicle or increasing doses of volasertib, and ECM gene transcripts were quantified by real-time PCR. Volasertib treatment resulted in dose-dependent reduction in the expression of *COL1A1, COL3A1, FN1*, and *ACTA2* (Fig. 5, A–D). Consistent with RT-PCR results, volasertib treatment reduced the protein levels of key ECM components, including COL1A1, COL3A1, ELN, and FN1, confirming its inhibitory effect on their expression at both the transcript and protein levels (Fig. 5, E–H).

**Figure 6.**
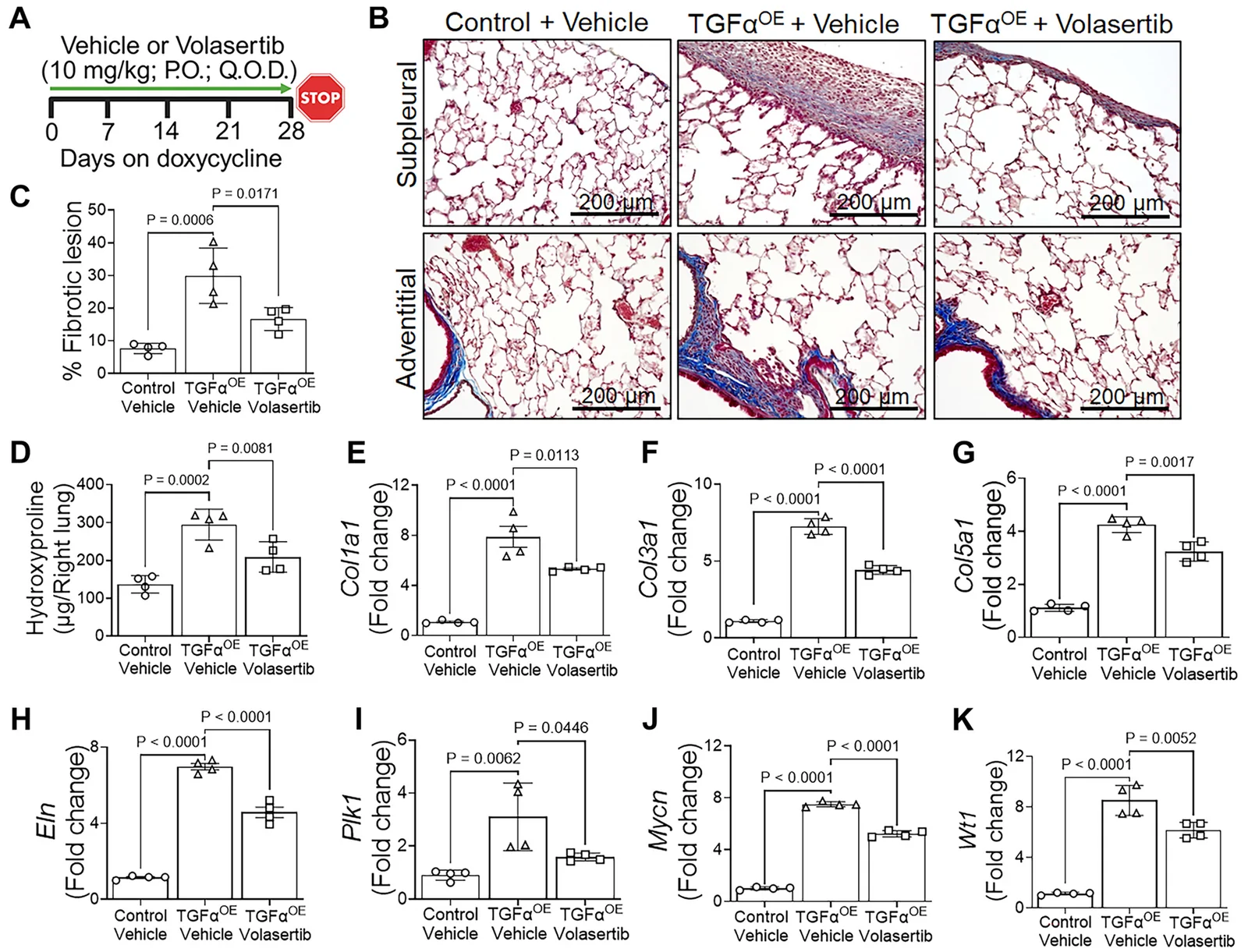
Volasertib attenuates TGFα-induced pulmonary fibrosis. *A*: schematic illustration of the volasertib treatment study. Control and TGFα mice were treated with either vehicle or volasertib (10 mg/kg; every alternate day via oral administration) for 4 wk, whereas they were fed with doxycycline-containing food and water. Panel *A* created with a licensed version of BioRender.com. *B*: representative images of Masson’s trichrome-stained lung sections from the vehicle or volasertib-treated mice. Images were obtained at 20× magnification. Scale bar, 200 μm. *C*: quantification of Masson’s trichrome-stained lung sections represented as percentage fibrotic area in mice treated with vehicle or volasertib (*n* = 2 male, *n* = 2 female; 1-way ANOVA followed by Tukey’s post hoc test). *D*: quantification of total lung hydroxyproline levels in mice treated with vehicle or volasertib (*n* = 2 male, *n* = 2 female; 1-way ANOVA followed by Tukey’s post hoc test). *E–K*: quantification of *Col1a1, Col3a1, Col5a1, Eln, Fn1, Acta2, Plk1, Mycn,* and *Wt1* gene transcripts in the total lung transcripts of mice treated with vehicle or volasertib (*n* = 2 male, *n* = 2 female; 1-way ANOVA followed by Tukey’s post hoc test). TGFα, transforming growth factor α.

To test if treatment with volasertib prevents the development of lung fibrosis, TGFα mice were concomitantly treated with DOX to induce TGFα expression and either volasertib (10 mg/kg body wt) or vehicle on alternate days for 4 wk (Fig. 6A). Induction of TGFα caused extensive subpleural, perivascular, and peribronchial fibrosis (Fig. 6B). TGFα-over-expressing mice that were concomitantly treated with volasertib showed reduced collagen staining, with infrequent, scattered, and small fibrotic areas. Furthermore, we observed a significant decrease in the fibrotic lesions and hydroxyproline levels in TGFα mice treated with volasertib compared with vehicle-treated mice (Fig. 6, C and D). In agreement with histological and biochemical changes, we observed a significant decrease in lung collagen (*Col1A1, Col3a1*, and *Col5A1*), and *Eln* levels in TGFα mice treated with volasertib compared with vehicle-treated TGFα mice on DOX for 4 wk (Fig. 6, E–H). Also, we observed that volasertib treatment attenuated the expression of *Plk1*, *Mycn*, and *Wt1* in TGFα overexpression mice (Fig. 6, I–K). These findings collectively suggest that pharmacological inhibition of PLK1 by volasertib attenuates fibroblast activation and pulmonary fibrosis.

## DISCUSSION

This study identifies the proto-oncogene MYCN as a positive regulator of fibroblast activation, including proliferation, survival, and ECM production in IPF fibroblasts. MYCN has also been implicated in the initiation and progression of several cancers, including neuroblastoma, medulloblastoma, Wilms’ tumor, and prostate, breast, and lung cancer (42–47). Our findings extend the functional significance of MYCN beyond oncogenesis, implicating it as a critical mediator of fibroblast activation and fibrotic remodeling during lung injury. Our findings suggest that MYCN is markedly upregulated in lung fibroblasts and fibroblastic lesions within the IPF lung, as well as in a murine model of TGFα-induced pulmonary fibrosis. Specifically, the profibrotic growth factor TGFα promotes MYCN expression by activating the transcription factor WT1, a known mediator of fibroblast activation (17, 34, 48, 49). A limitation of the current study is that the human lung explants used were predominantly derived from male donors, which may limit the generalizability of our findings regarding sex-specific differences in pulmonary fibrosis. Therefore, future studies are warranted to validate MYCN upregulation in fibroblasts using more diverse and sex-balanced IPF patient cohorts.

Using knockdown studies, we identified MYCN-driven gene networks that were fibroblast-specific and dysregulated in IPF, such as genes involved proliferation (*PLK1, CDK1, CCNE1*, and *AURKB*), survival (*BCL2* and *BAD*), and ECM production (*COL1A1, COL3A1, ELN*, and *FN1*). Supporting its profibrotic role, loss of MYCN resulted in reduced fibroblast proliferation and enhanced apoptotic clearance, indicating that MYCN acts as a positive regulator of fibroblast activation. This is consistent with previous findings, where MYCN has been shown to promote cell proliferation and survival during tumor progression across multiple cancer types (43, 50–52).

MYCN has been shown to regulate PLK1, a critical cell cycle regulator that drives fibroblasts proliferation (29, 53). Published studies in neuroblastoma and small cell lung carcinoma models demonstrate that MYCN and PLK1 form a feedforward regulatory loop, in which MYCN upregulates PLK1 expression, and PLK1, in turn, stabilizes MYCN. This reciprocal interaction sustains elevated PLK1 levels, contributing to uncontrolled proliferation and tumor progression (27, 28). Our findings extend this paradigm by showing that disruption of the PLK1-MYCN feedback loop by volasertib not only suppresses MYCN but also downregulates WT1 expression. This highlights a previously unrecognized axis in fibrotic fibroblasts and suggests that targeting PLK1 may attenuate fibroblast activation through simultaneous inhibition of MYCN- and WT1-driven profibrotic programs.

Volasertib is a known PLK1 inhibitor that binds to the ATP-binding pocket of PLK1 and thereby inhibits PLK1 enzymatic activity essential for cell division and proliferation (54, 55). Our findings indicate that pharmacological inhibition of PLK1 with volasertib suppresses the expression of MYCN and WT1, key transcriptional drivers of enhanced proliferation and survival in IPF fibroblasts. Volasertib treatment in vivo attenuated the profibrotic gene expression (*Col1a1, Col3a1, Col5a1, Eln*, and *Fn1*) and collagen deposition in the lungs during TGFα-induced pulmonary fibrosis. The protective effects of volasertib are comparable with our previous findings with the FDA-approved antifibrotic agent nintedanib, which attenuated fibroblast activation, including proliferation, survival, and collagen deposition, in this model (11, 22, 31). However, future studies are warranted to determine whether volasertib overlaps with or acts synergistically with existing antifibrotic therapies, such as nintedanib and pirfenidone, in attenuating fibroblast activation in IPF. A recent study by Van den Bossche et al. (56) demonstrated that volasertib exerts potent antiproliferative effects in non-small-cell lung cancer, primarily through PLK1 inhibition-induced apoptosis. This phenomenon was also documented in multiple cancer types, including neuroblastoma, renal cell carcinoma, anaplastic thyroid cancer, and leukemia (27, 28, 50, 57, 58). In cancer cells, volasertib acts primarily by blocking PLK1 activity, leading to mitotic arrest, mitotic catastrophe, and apoptosis, while also attenuating transcriptional programs driven by oncogenic factors such as MYCN. This dual impact on both cell cycle progression and transcriptional regulation positions volasertib as a mechanistically distinct and promising therapeutic strategy for fibroblast-driven diseases such as IPF.

Our study provides evidence for the functional role of MYCN in regulating fibroblast proliferation, apoptotic resistance, and extracellular matrix production through the WT1-MYCN-PLK1 axis. Nonetheless, several limitations remain. Future studies are required to determine whether WT1 directly binds to the MYCN promoter to establish direct transcriptional regulation. In addition, in vivo studies using fibroblast-specific MYCN conditional deletion models, such as TBX4 or PDGFRα promoter-driven Cre-recombinase mice, will be critical to define the cell-autonomous contribution of MYCN to fibroblast activation and pulmonary fibrosis.

In summary, we have identified and described the pathological role of MYCN and PLK1 in the proliferation and survival of IPF fibroblasts. Pharmacological inhibition of PLK1 with volasertib represents a promising therapeutic avenue to mitigate transcriptional programs underlying fibroblast activation and ECM deposition in IPF. Our preclinical findings support the use of volasertib as an antifibrotic agent to attenuate fibroblast activation involved in severe fibrotic lung disease.

### Perspectives and Significance

Pulmonary fibrosis remains a devastating disease with limited therapeutic options and poor prognosis. Understanding the molecular mechanisms driving fibroblast activation is essential for developing effective therapeutic interventions. In this study, we uncovered a previously unrecognized role for MYCN and PLK1 as key positive regulators of fibroblast proliferation, survival, and extracellular matrix production. Importantly, we demonstrate that WT1 drives MYCN expression, thereby establishing a WT1-MYCN-PLK1 signaling axis that promotes fibroblast activation. These findings not only provide new mechanistic insights into fibroblast biology in pulmonary fibrosis but also highlight this signaling pathway as a promising therapeutic target to mitigate disease progression.

## Supplementary Material

Supplementary Material

Supplemental Figs. S1–S5: https://doi.org/10.6084/m9.figshare.30019093.

## Figures and Tables

**Table 1.** The list of human RT-PCR primers used in the study

Gene Symbol	Forward Primer	Reverse Primer
*ACTA2*	GCTTTCAGCTTCCCTGAACA	GGAGCTGCTTCACAGGATTC
*ACTNB*	CCAACCGCGAGAAGATGA	CCAGAGGCGTACAGGGATAG
*AURKB*	GATGGCCCAGAAGGAGAACT	AGGCTCTTTCCGGAGGACT
*BAD*	CCCAGAGTTTGAGCCGAGTG	CCCATCCCTTCGTCGTCCT
*BCL2*	AGTACCTGAACCGGCACCT	GCCGTACAGTTCCACAAAGG
*CCNE1*	GGGACACCATGAAGGAGGA	TCTTCATCTGGATCCTGCAA
*CDK1*	TGGATCTGAAGAAATACTTGGATTCTA	CAATCCCCTGTAGGATTTGG
*COL1A1*	GGGATTCCCTGGACCTAAAG	GGAACACCTCGCTCTCCA
*COL3A1*	TGGTGGTAAAGGCGAAATG	AGTCCAGGAGCACCATTAGC
*FN1*	CTGGCCGAAAATACATTGTAAA	CCACAGTCGGGTCAGGAG
*LOXL2*	GGAGAGGACATACAATACCAAAGTGT	CCATGGAGAATGGCCAGTAG
*MYCN*	TGATCCTCAAACGATGCCTTC	GGACGCCTCGCTCTTTATCT
*PLK1*	AACGACTTCGTGTTCGTGGT	AGGGCTTTCCTCCTCTTGTG
*WT1*	AGCTGTCCCACTTACAGATGC	CCTTGAAGTCACACTGGTATGG

*ACTA2*, smooth muscle alpha-2 actin; *AURKB*, Aurora kinase B; *BAD*, BCL2-associated agonist of cell death; *BCL2*, B-cell leukemia/lymphoma 2; *CCNE1*, cyclin E1; *CDK1*, cyclin-dependent kinase 1; *COL1A1*, collagen 1; *COL3A1*, collagen 3; *FN1*, fibronectin 1; *LOXL2*, lysyl oxidase homolog 2; *MYCN*, N-myc proto-oncogene; *PLK1*, Polo-like kinase 1; *WT1*, Wilms’ tumor 1.

**Table 2.** The list of mouse RT-PCR primers used in the study

Gene Symbol	Forward Primer	Reverse Primer
*Acta2*	TGACGCTGAAGTATCCGATAGA	CGAAGCTCGTTATAGAAAGAGTGG
*Col1a1*	AGACATGTTCAGCTTTGTGGAC	GCAGCTGACTTCAGGGATG
*Col5a1*	AATGGGTGACCCTGGAGAA	CCATGGGGCCCTTAGAAC
*Col3a1*	TCCCCTGGAATCTGTGAATC	TGAGTCGAATTGGGGAGAAT
*Eln*	TGGAGCAGGACTTGGAGGT	CCTCCAGCACCATACTTAGCA
*Hprt*	GCCCTTGACTATAATGAGTACTTCAGG	TTCAACTTGCGCTCATCTTAGG
*Mycn*	AGCACCTCCGGAGAGGATAC	CCACATCGATTTCCTCCTCT
*Plk1*	TTGTAGTTTTGGAGCTCTGTCG	CAGTGCCTTCCTCCTCTTGT
*Wt1*	CAGATGAACCTAGGAGCTACCTTAAA	TGCCCTTCTGTCCATTTCA

*Acta2*, smooth muscle alpha-2 actin; *Col1a1*, collagen 1; *Col3a1*, collagen 3; *Col5a1*, collagen 5; *Eln*, elastin; *Hprt*, hypoxanthine-guanine phosphoribosyl transferase; *Mycn*, N-myc proto-oncogene; *Plk1*, Polo-like kinase 1; *Wt1*, Wilms’ tumor 1.

**Table 3.** The list of antibodies and their dilutions used for immunohistochemistry and Western blotting

Target	Dilution		Clone	Cat. No.	Source	RRID	Reference
	IHC	WB					
αSMA		1:20,000	1A4	A5228	Sigma	AB_262054	Gajjala et al. (34)
BCL2		1:1,000		3498	Cell Signaling Technology	AB_1903907	Ediga et al. (19)
COL1A1		1:1,000		91144	Cell Signaling Technology	AB_2800169	Yombo et al. (35)
COL3A1		1:1,000		66887	Cell Signaling Technology		Shuai et al. (36)
ELN		1:1,000		15257-1-AP	Proteintech	AB_2878120	Yombo et al. (35)
FN1		1:2,000	FBN11	MA5-11981	Invitrogen	AB_10982280	Zhang et al. (37)
GAPDH		1:5,000		A300-643A	Bethyl Laboratories		Ediga et al. (19)
MYCN		1:1,000	NCM II 100	OP-13	Millipore	AB_2266879	Sabo et al. (38)
MYCN	1:500		B8.4.b	sc-53993	Santa Cruz	AB_831602	Tao et al. (39)
PLK1		1:2,000	35-206	37-7000	Thermo Fischer Scientific	AB_2533335	Yu et al. (40)
WT1		1:500		12609-1-AP	Proteintech	AB_2216225	Ediga et al. (19)
Rabbit IgG (HRP-linked)		1:10,000		7074	Cell Signaling Technology	AB_2099233	Ediga et al. (19)
Mouse IgG (HRP-linked)		1:20,000		7076	Cell Signaling Technology	AB_330924	Ediga et al. (19)
Mouse IgG2A (biotin)				115065206	Jackson ImmunoResearch	AB_2338572	Gajjala et al. (34)

αSMA, alpha-smooth muscle actin; BCL2, B-cell lymphoma 2; COL1A1, collagen 1; COL3A1, collagen 3; ELN, elastin; FN1, fibronectin 1; GAPDH, glyceraldehyde-3-phosphate dehydrogenase; HRP, horseradish peroxidase; MYCN, N-myc proto-oncogene; PLK1, Polo-like kinase 1; WT1, Wilms’ tumor 1.

## Data Availability

The data that support the findings of this study are available on request from the corresponding author. The data are not publicly available due to privacy or ethical restrictions.
