# Supplementary material for: PLK1 inhibition by volasertib suppresses key transcriptional regulators underlying fibroblast activation and pulmonary fibrosis
Source: Am J Physiol Lung Cell Mol Physiol. Author manuscript; Available in PMC 2026 Sep 1. (PMC13531454; doi:10.1152/ajplung.00204.2025)
Supplement: Supplementary file 1 [file NIHMS2201664-supplement-Supplementary_Material.pdf]

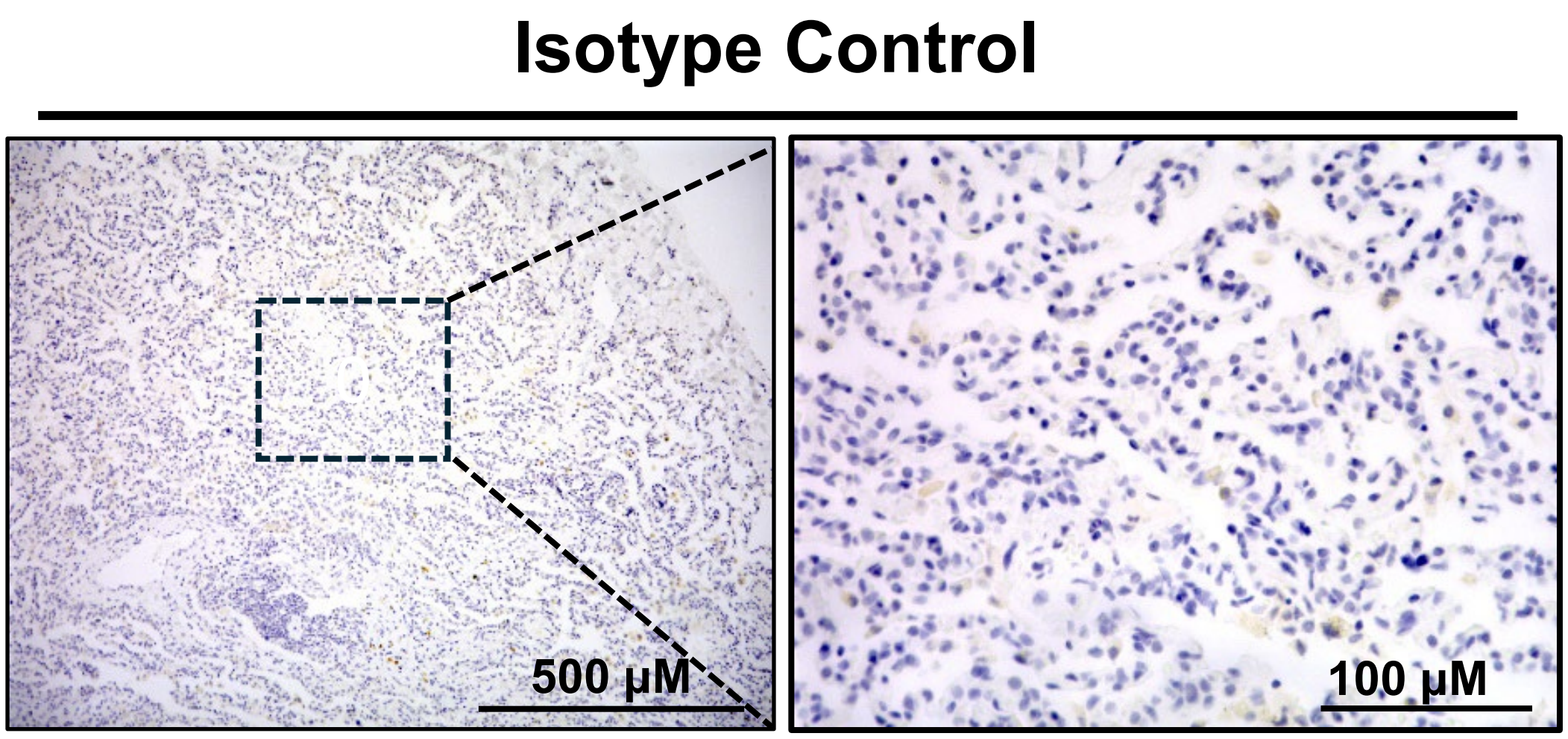

Supplementary Figure 1. Representative images of distal lung section at both lower and higher magnification immuno-stained with isotype control (IgG2A) antibody.

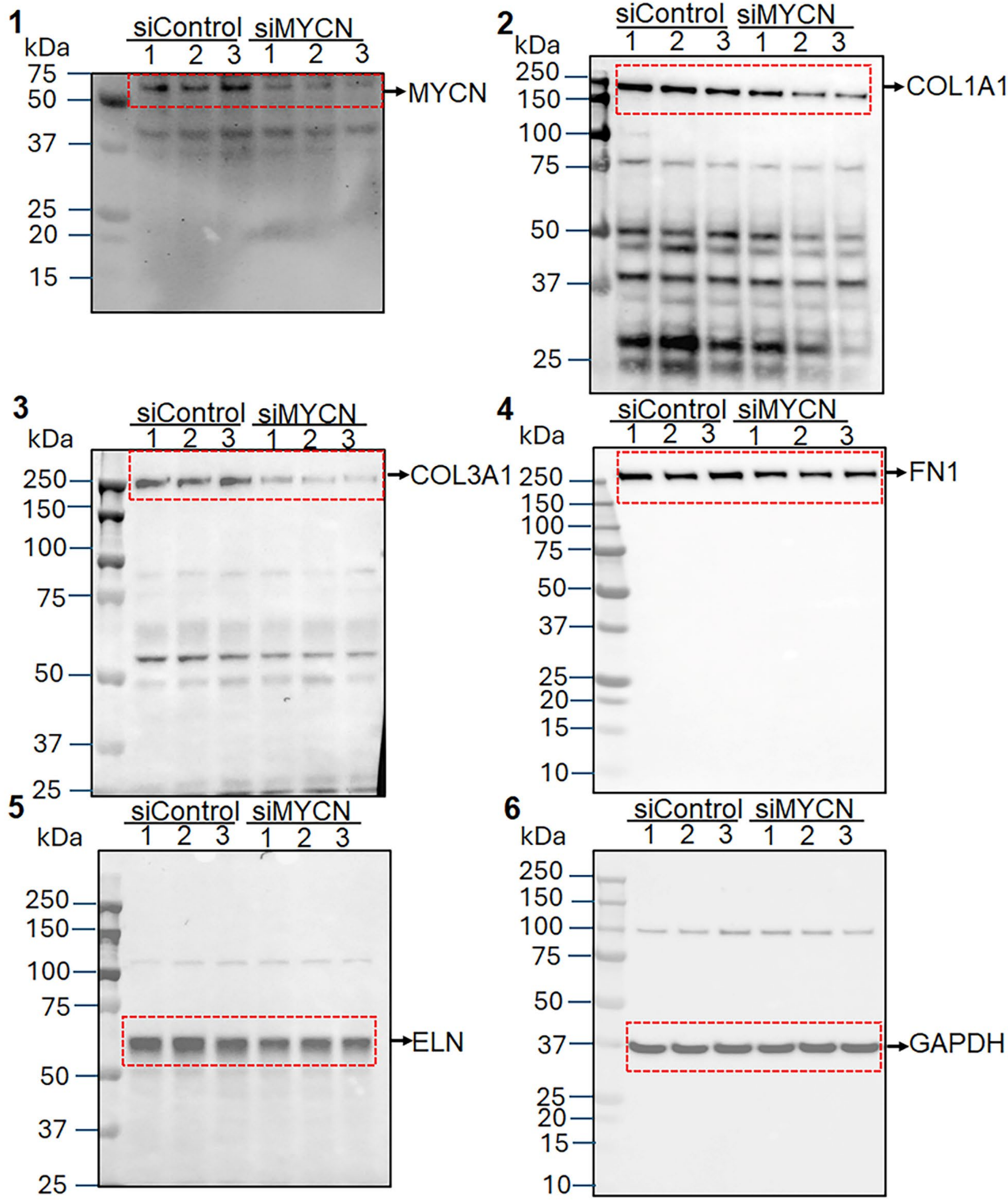

Supplementary Figure 2. Full blot images for Figure 2G

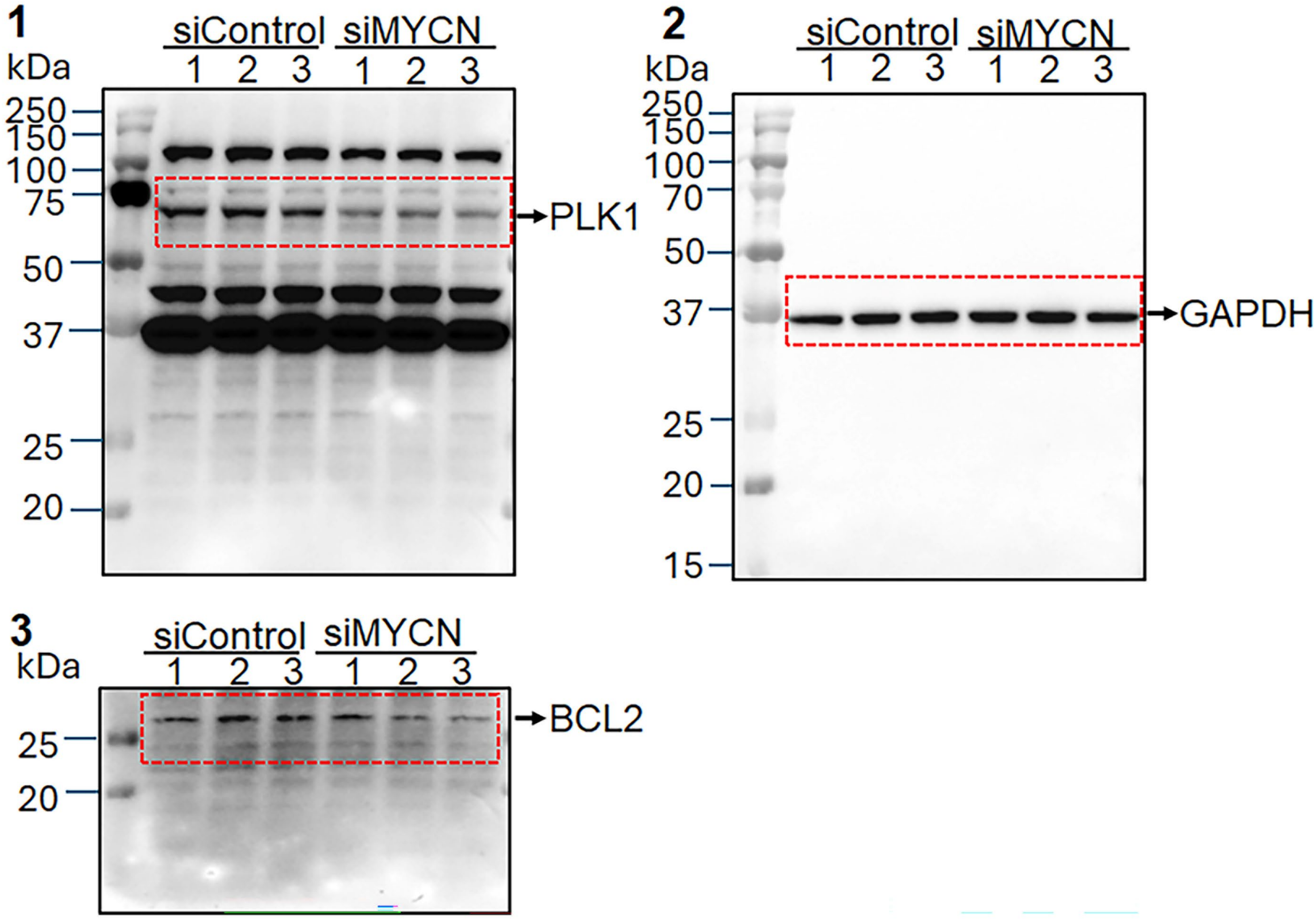

Supplementary Figure 3. Full blot images for Figure 3G

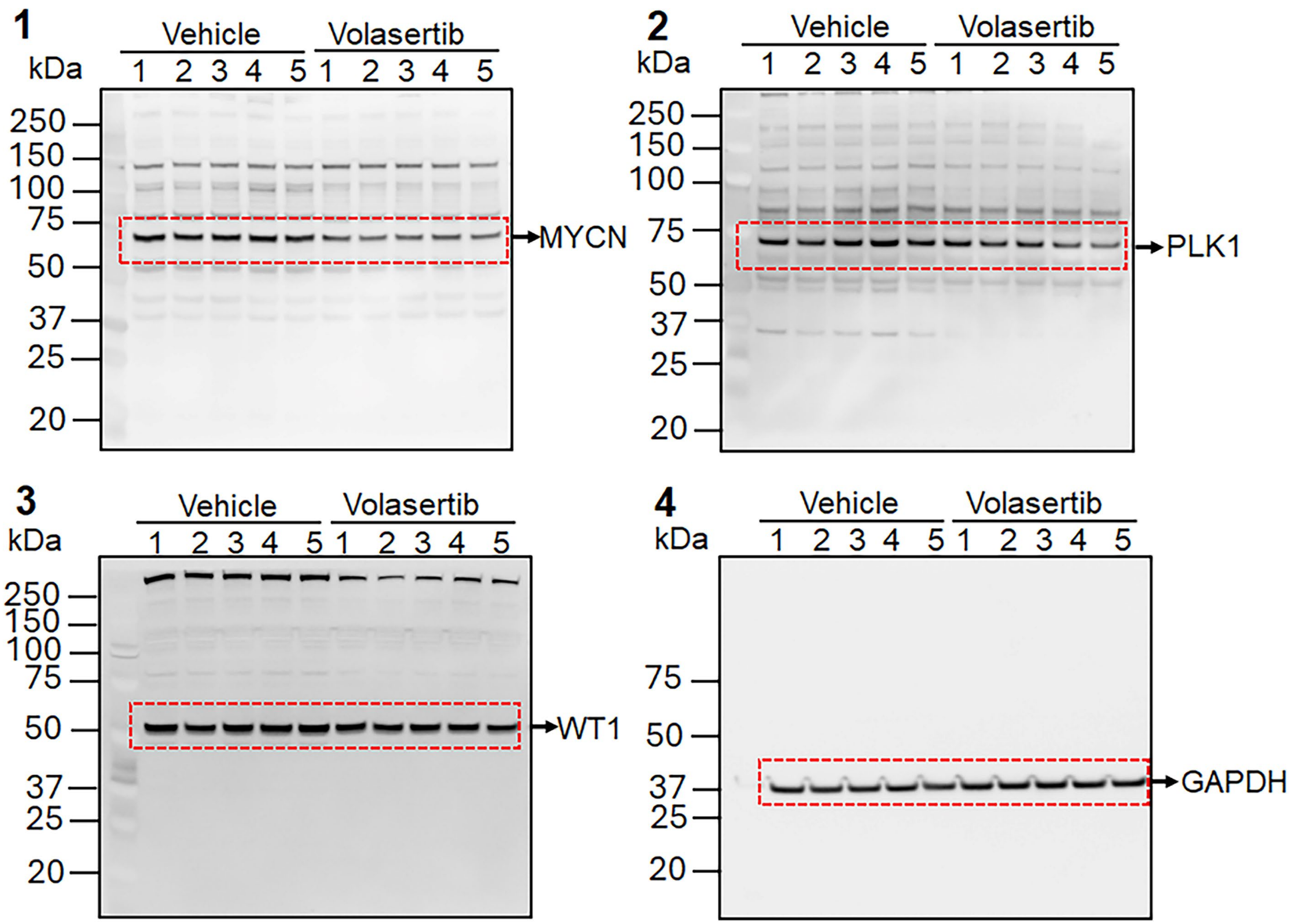

Supplementary Figure 4. Full blot images for Figure 4A

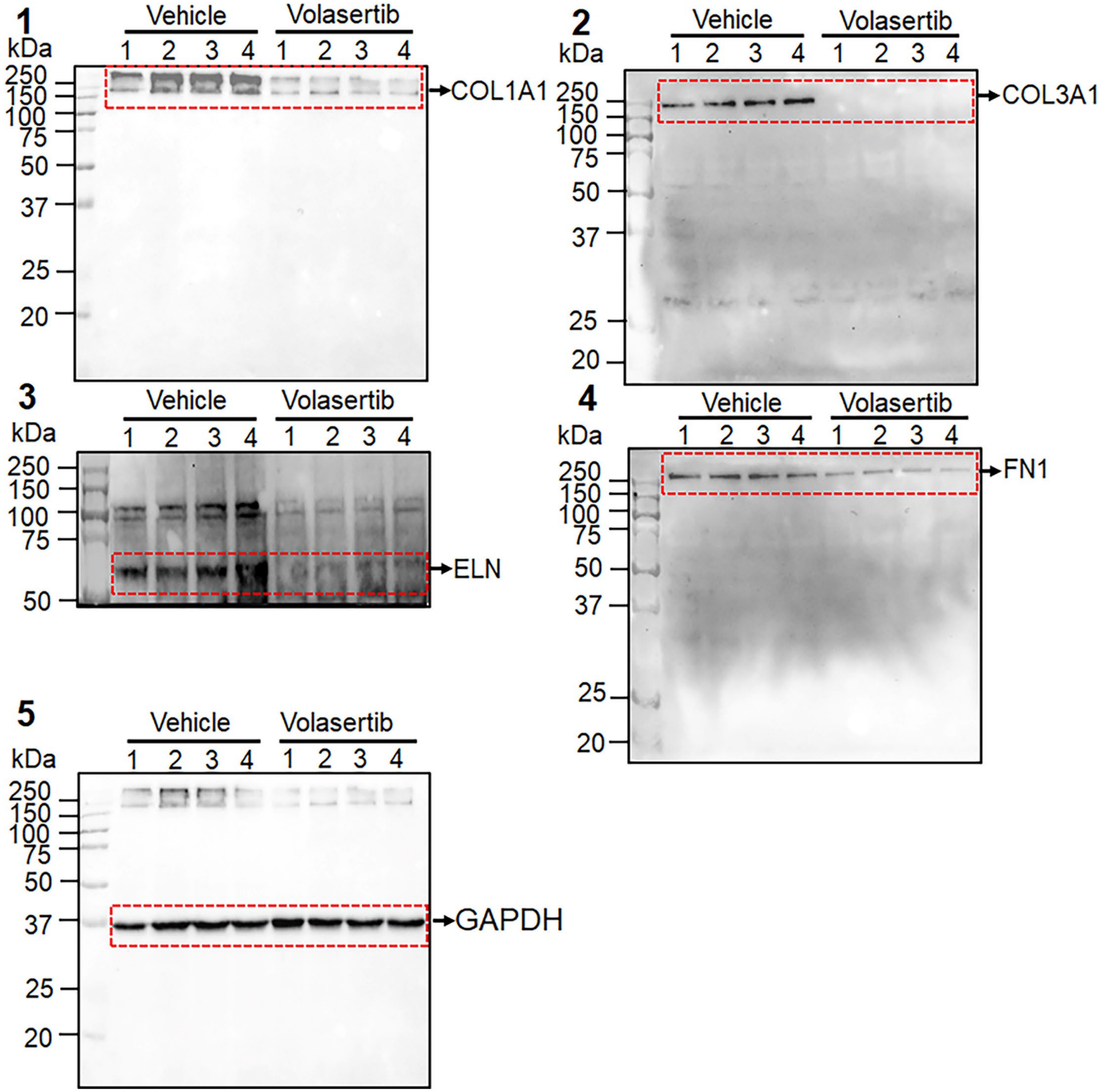

Supplementary Figure 5. Full blot images for Figure 5E
